# Factors associated with the consultation of GPs among adults aged ≥16 years: an analysis of data from the Health Survey for England 2019

**DOI:** 10.3399/BJGPO.2022.0177

**Published:** 2023-07-26

**Authors:** Fujian Song, Max Bachmann, Amanda Howe

**Affiliations:** 1 Faculty of Medicine and Health Sciences, Norwich Medical School, University of East Anglia, Norwich, UK

**Keywords:** general practice, general practitioners, consultation frequency, sociodemographic factors

## Abstract

**Background:**

Understanding the factors associated with demands for general practice care is crucial for policy decision makers to appropriately allocate healthcare resources.

**Aim:**

To investigate factors associated with the frequency of GP consultations.

**Design & setting:**

Data on 8086 adults aged ≥16 years was obtained from cross-sectional Health Survey for England (HSE) 2019.

**Method:**

The primary outcome was the frequency of consultations of a GP in the last 12 months. Multivariable ordered logistic regression analysis was used to evaluate associations between GP consultations and a range of sociodemographic and health-related factors.

**Results:**

Frequency of GP consultations for all reasons was higher among females (odds ratio [OR] 1.81, 95% confidence interval [CI] = 1.64 to 2.01), those aged ≥75 years (OR 1.48, 95% CI = 1.15 to 1.92), ethnic minority populations (Black: OR 1.42, 95% CI = 1.09 to 1.84; Asian: OR 1.53, 95% CI = 1.25 to 1.87), lowest household income (OR 1.53, 95% CI = 1.29 to 1.83), adults with long-lasting illnesses (OR 3.78, 95% CI = 3.38 to 4.22), former smokers (OR 1.17, 95% CI = 1.04 to 1.22), being overweight (OR 1.14, 95% CI = 1.01 to 1.29), and being obese (OR 1.32, 95% CI = 1.16 to 1.50). Predictors of consultations for physical health problems were similar to predictors of consultations for any health problems. However, younger age was associated with more consultations for mental health problems, or a combination of mental and physical health problems.

**Conclusion:**

The higher frequency of consultation of GPs is associated with female sex, older age, ethnic minority populations, being socioeconomically disadvantaged, existence of lasting illnesses, smoking, being overweight, and being obese. Older age is associated with increased consultations for physical health problems, but associated with reduced consultations for mental health or a combination of mental and physical health problems.

## How this fits in

NHS payments to general practices in England are currently based on assumed determinants of demand, including age, sex, patient need (morbidity and mortality), list turnover, market forces, rurality, and patients in nursing or residential homes. While most previous studies used medical records data from the Clinical Practice Research Datalink (CPRD), this study used data from HSE 2019. The results confirmed that the higher frequency of consultation of GPs is associated with female sex, older age, ethnic minority populations, being socioeconomically disadvantaged, existence of lasting illnesses, smoking, being overweight, and being obese. Although older age was associated with more frequent GP consultations for physical health problems, younger age was associated with relatively more frequent consultations for mental health problems.

## Introduction

Demand for general practice services has been increasing in the UK.^1^ Because of increased workload and pressure, many general practice staff have left their posts, worsening the shortage of GPs.^2^ Patients are encouraged to attend to understand the diagnosis underlying their symptoms and to find out how to manage their problems in order to make early diagnoses and treatment, and to assist self-care; this may contribute to increased requests for appointments. However, pressure on general practice services needs to be appropriately managed.

Previous studies have found that consultation rates in general practice were higher among older patients and females, among more socioeconomically deprived patients, people from an Asian ethnic group, and people currently smoking.^3–5^ NHS payments to general practices in England are based on assumed determinants of demand, including age and sex, patient need (morbidity and mortality), list turnover, rurality, and residence in nursing or residential homes.^6^ A detailed understanding of factors associated with needs and demands for general practice care is crucial for policy decision makers to appropriately allocate healthcare resources and to improve the sustainability of general practice services. This study provides further evidence on sociodemographic and health-related factors associated with the use of GPs, using data from HSE 2019.

## Method

### Data source

Data from HSE 2019 were used, which had a cross-sectional design and provided information on health of a sample of adults and children living in private households in England.^7^ The survey data included specific health conditions and risk factors, indicators of socioeconomic position, and use of healthcare services. Of a total of 8205 adults (aged ≥16 years) interviewed, 8086 provided data on their frequency of use of general practice services.

### Outcome and variables

The primary outcome was the number of times a patient consulted a GP in the last 12 months.^8^ The question was *'In the last 12 months, approximately how many times talked to, or visited a GP or family doctor about your own health?'* Possible responses were five numerically ordered groups: 0; 1 or 2; 3–5; 6–10; or >10 times. Consultations were further categorised as consultations for physical health problems, for mental health problems, or for both physical and mental health problems (see Supplementary Box S1).

The following explanatory variables were investigated: sex; age; Index of Multiple Deprivation; equivalised household income; highest educational qualification (HEQ); rurality; smoking status; weekly alcohol consumption; body mass index (BMI); and the existence of any physical or mental health conditions or illnesses lasting ≥12 months. All explanatory variables were categorical. The Index of Multiple Deprivation was categorised by quintile from the least deprived to the most deprived (quintile Index of Multiple Deprivation; QIMD). Similarly, the equivalised household income was grouped by quintile from the highest to the lowest (quintile equivalised household income; QEHI). The highest educational qualification was categorised into three groups: degree or equivalent; below degree; and no educational qualification. More details on the outcome and explanatory variables are available in Supplementary Box S1.^9^

### Statistical analysis methods

Associations between the dependent and explanatory variables were estimated using ordered logistic regression analysis (Stata/MP version 17.0). The dependent variable was the number of times a GP was consulted in the last 12 months, categorised as above. The study also separately conducted analyses of GP consultations for physical health problems only, and GP consultations for any mental health problems, which included consultations for mental health or a combination of mental and physical health problems. The analyses of GP consultations for physical health problems excluded participants who consulted a GP for any mental health problems, and the analyses of consultations for any mental health problems excluded participants who consulted a GP for physical health problems only.

A proportional odds model was adopted for ordered logistic regression analysis, which assumed that a common OR represented the association of each explanatory variable with the ordinal outcome. The proportional odds assumption was not statistically tested, because the null hypothesis can be incorrectly rejected.^10,11^ Each explanatory variable had at least two categories, and one was used as the reference category. Compared with the reference category, OR = 1 indicates no association between a factor and GP consultations, OR >1 indicates that a factor is associated with more GP consultations, and OR <1 indicates that a factor is associated with reduced GP consultations. Statistical significance was defined as two-sided *P*-value <0.05. An OR is statistically significant (*P*<0.05) if OR =1 is not contained within its 95% CI. The Wald statistical test of joint null hypotheses was also conducted that OR = 1 for all categories of an explanatory variable.

Univariable analyses were first conducted in which the dependent variable and a single explanatory variable were involved in the model. Then multivariable analyses were conducted using explanatory variables that were statistically significant in the univariable analyses. To obtain parsimonious models (that is, models with as few explanatory variables as necessary), multivariable analyses were conducted after manually excluding statistically non-significant variables. Because age, sex, rurality, and lasting illnesses currently determine payments to general practices in England,^6^ these explanatory variables were included in all the models regardless of their statistical significance.

All univariable and multivariable analyses were weighted using the interview weights for HSE 2019 (see Supplementary Box S1 for more details on the interview weights).^9^ Statistical analyses were conducted after excluding participants with missing data. Sensitivity analysis was conducted that included participants with missing data, by creating a category of ‘unknown’ for missing data on explanatory variables. Sensitivity analyses were also conducted regarding the impact of lasting illnesses, and different measures of socioeconomic status.

## Results

Table 1 shows the main characteristics of participants included in this study. Of the 8086 participants, 55.5% were female and 44.5% were male. In terms of ethnic group, 85.7% were White, 3.0% were Black, 8.6% were Asian, and 2.7% were other or unknown ethnic minorities. The highest educational qualification was degree or equivalent for 28.8%, below degree for 50.4%, and no qualification for 20.3% of participants. It was reported that 37.2% of participants consulted a GP once or twice, 23.3% 3–5 times, 9.9% 6–10 times, and 6.8% >10 times in the last 12 months (Table 1).

**Table 1.** The main characteristics of study participants

**Variable**	***n***	**%**
**Total**	8086	100
**Sex**		
Male	3596	44.5
Female	4490	55.5
**Age groups, years**		
16–24	702	8.7
25–34	1037	12.8
35–44	1379	17.1
45–54	1406	17.4
55–64	1341	16.6
65–74	1236	15.3
≥75	985	12.2
**Ethnic group**		
White	6928	85.7
Black	240	3.0
Asian	697	8.6
Other	221	2.7
**Quintile Index of Multiple Deprivation**		
1 (least deprived)	1671	20.7
2	1556	19.2
3	1575	19.5
4	1607	19.9
5 (most deprived)	1677	20.7
**Quintile equivalised household income**		
Highest	1215	15.0
Second	1439	17.8
Third	1334	16.5
Fourth	1325	16.4
Lowest	1196	14.8
Unknown	1577	19.5
**Highest educational qualification**		
Degree or equivalent	2329	28.8
Below degree	4075	50.4
None	1638	20.3
Unknown	44	0.5
**Rurality**		
Urban	6555	81.1
Rural	1531	18.9
**Lasting illnesses (>12 months)**		
No	4382	54.2
Yes	3698	45.7
Unknown	6	0.1
**Smoking status**		
Never	4750	58.7
Former	2066	25.6
Current	1221	15.1
Unknown	49	0.6
**Weekly alcohol consumption**		
Non-drinker	1540	19.0
Lower risk	4550	56.3
Increasing risk	1473	18.2
Higher risk	333	4.1
Unknown	190	2.3
**Body mass index**		
Normal	2455	30.4
Overweight	2723	33.7
Obesity	2230	27.6
Underweight	96	1.2
Unknown	582	7.2
**Times consulted a GP in the last 12 months**		
0	1846	22.8
1 or 2	3004	37.2
3–5	1887	23.3
6–10	797	9.9
>10	552	6.8

In the parsimonious multivariable model, number of times consulted a GP were positively and independently associated with female sex (OR 1.81, 95% CI = 1.64 to 2.01), those aged ≥75 years (OR 1.48, 95% CI = 1.15 to 1.92), ethnic minority populations (Black: OR 1.42, 95% CI = 1.09 to 1.84; Asian: OR 1.53, 95% CI = 1.25 to 1.87), lowest household income (OR 1.53, 95% CI = 1.29 to 1.83), adults with long-lasting illnesses (OR 3.78, 95% CI = 3.38 to 4.22), former smokers (OR 1.17, 95% CI = 1.04 to 1.22), being overweight (OR 1.14, 95% CI = 1.01 to 1.29), and being obese (OR 1.32, 95% CI = 1.16 to 1.50) (Table 2).

**Table 2.** Association between number of times a GP was consulted for all reasons and related factors using multivariable ordered logistic regression models

**Variable**	**OR (95% CI**)	***P*-value^a^**
**Sex**		<0.001
Male	1.00	
Female	1.81 (1.64 to 2.01)	
**Age, years**		0.057
16–24	1.00	
25–34	1.13 (0.89 to 1.44)	
35–44	1.09 (0.87 to 1.36)	
45–54	1.13 (0.90 to 1.41)	
55–64	1.21 (0.96 to 1.52)	
65–74	1.25 (0.99 to 1.58)	
≥75	1.48 (1.15 to 1.92)	
**Ethnic group**		<0.001
White	1.00	
Black	1.42 (1.09 to 1.84)	
Asian	1.53 (1.25 to 1.87)	
Other	1.40 (1.02 to 1.92)	
**Quintile equivalised household income**		<0.001
Highest	1.00	
Second	1.14 (0.98 to 1.32)	
Third	1.13 (0.97 to 1.33)	
Fourth	1.33 (1.13 to 1.57)	
Lowest	1.53 (1.29 to 1.83)	
**Rurality**		0.291
Urban	1.00	
Rural	0.94 (0.83 to 1.06)	
**Lasting illnesses (>12 months)**		<0.001
No	1.00	
Yes	3.78 (3.38 to 4.22)	
**Smoking status**		0.029
Never regular	1.00	
Former	1.17 (1.04 to 1.32)	
Current	1.04 (0.88 to 1.22)	
**Body mass index**		<0.001
Normal	1.00	
Overweight	1.14 (1.01 to 1.29)	
Obesity	1.32 (1.16 to 1.50)	
Underweight	0.72 (0.42 to 1.23)	

The number of observations was 6096 in the analysis. Analysis was weighted using the interview weights for Health Survey England 2019. OR >1 indicates more frequent GP consultations in the higher ranked categories, and vice versa. ^a^ *P*-values were testing of the joint null hypotheses that OR = 1 for all categories of an explanatory variable. OR = odds ratio.

Results of univariable ordered logistic regression analyses showed that higher GP consultations were statistically significantly associated with female sex, older age, ethnic minority, the most deprived, lack of educational qualification, lower household income, lasting illnesses, former smokers, and being overweight or obese (see Supplementary Table S1). In the parsimonious multivariable model, number of times consulted a GP were positively and independently associated with female sex, aged ≥75 years, ethnic minority populations, lowest household income, lasting illnesses, former smokers, being overweight, and being obese (Table 2). Rurality was not statistically significantly associated with the frequency of consultations (OR 0.94, 95% CI = 0.83 to 1.06; *P* = 0.291). Lasting illnesses had the strongest independent association with GP consultations (OR 3.78, 95% CI = 3.38 to 4.22; *P*<0.001).

### Reasons for consultations

**Figure 1.**
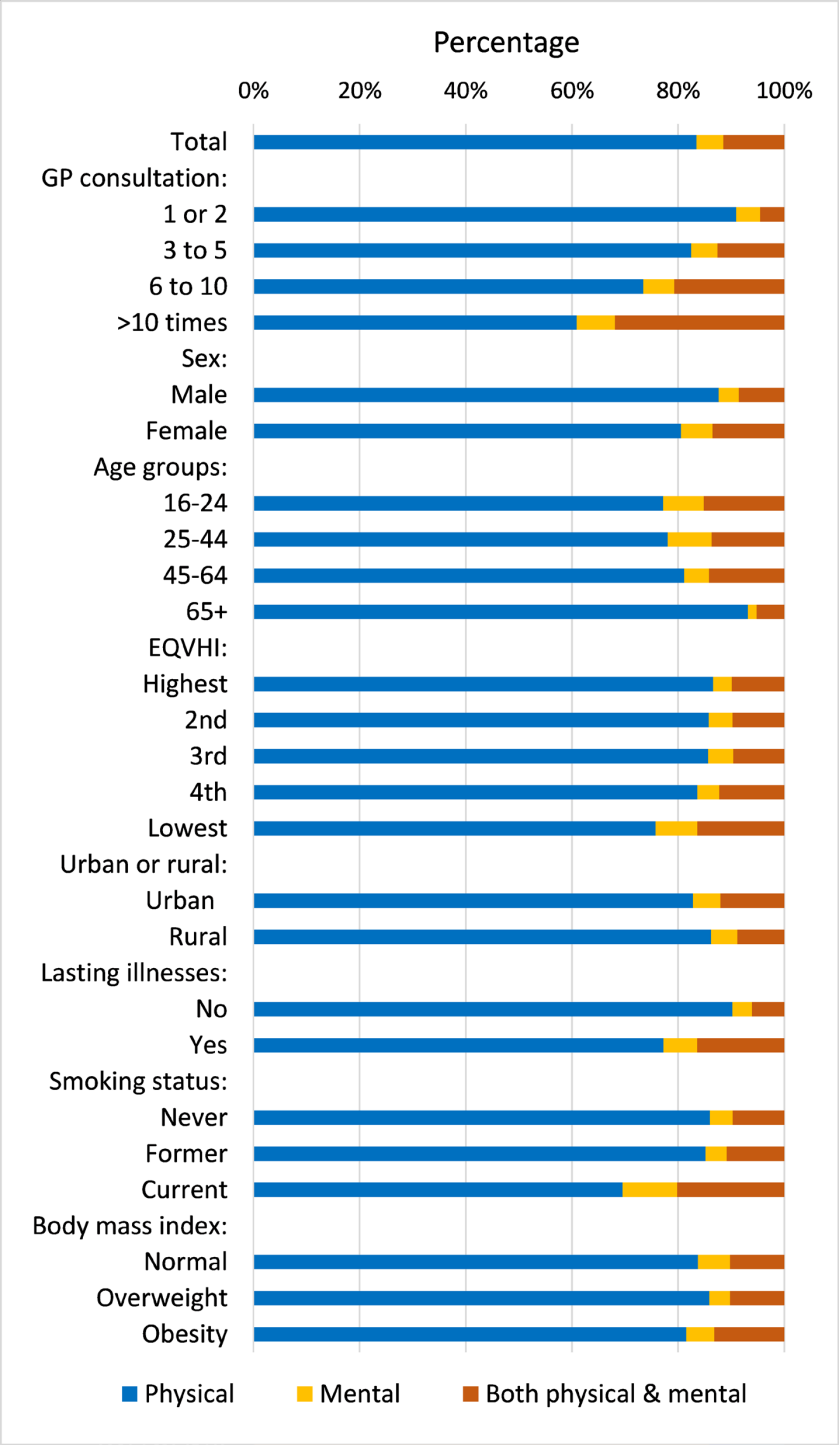
Distribution of reasons for GP consultations by participant characteristics. EQVHI = quintile equivalised household income.

Regarding reasons for GP consultations, 83.5% were for physical health problems, 5.1% for mental health problems, and 11.5% for a combination of physical and mental health problems (Figure 1). The proportion of consultations for any mental health problems was more common in more frequent users, from 9.1% among participants who visited a GP once or twice to 39.1% among those who visited a GP >10 times in the last 12 months. Consultations for mental health problems also tended to be higher for females, younger age groups, those who were socioeconomically disadvantaged, adults with lasting illnesses, and current smokers.

**Figure 2.**
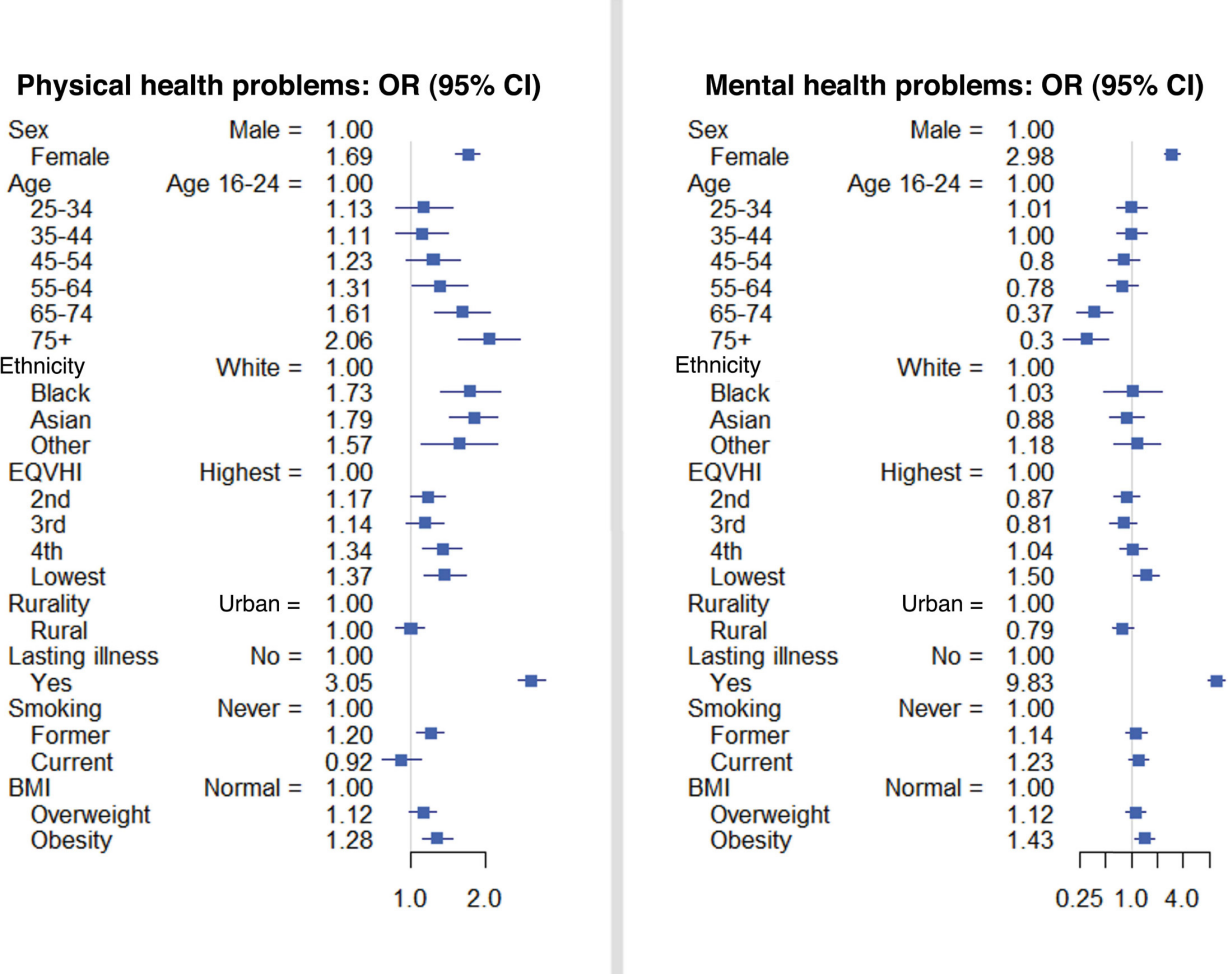
Factors associated with GP consultations for physical or mental health problems: results of multivariable ordered logistic regression analyses. The number of participants was 5320 and 2195, respectively, in the analysis of GP consultations for physical only and any mental health problems, including 1419 non-users in both analyses. BMI = body mass index. EQVHI = equivalised household income quintile.

Results of the separate multivariable analyses for physical health only and for any mental health problems are reported in Figure 2 and Supplementary Table S2. The results of multivariable analysis for physical health problems were generally similar to the analyses for all consultations; however, younger age was associated with more consultations for any mental health problems, contrasting to the positive association between the older age and higher GP consultations for physical health problems. Compared with people who never smoked, former smokers were associated with increased frequency of GP consultations for physical health problems (OR 1.20, 95% CI = 1.06 to 1.36), while current smoker tended to have more GP consultations for mental health problems (OR 1.23, 95% CI = 0.94 to 1.61). In all multivariable analyses, the independent variable most strongly associated with consultation frequency was lasting illnesses (ORs 3.78, 3.05, and 9.83, respectively, for all, physical health only, and any mental health problems).

### Sensitivity analyses

Sensitivity analyses were conducted in which participants with missing data were included in the analysis by creating a category of ‘unknown’ for missing data on explanatory variables. The number of participants increased from 6096 to 8086 after including participants with missing data in the analysis. Results of analysis including missing data were mostly identical to the results of the analysis excluding the missing data (see Supplementary Table S3). However, weekly alcohol consumption was retained in the model when missing data were utilised. Weekly alcohol consumption <14 units was associated with lower GP consultations, compared with non-drinkers.

The existence of conditions or illnesses lasting ≥12 months is the most strongly associated with consultation frequency, and it is likely also to be associated with other explanatory variables. After excluding lasting illnesses from the model, to estimate the direct effects of the other variables not mediated through lasting illnesses, the association between older age and the times a GP was consulted became much stronger and more statistically significant (see Supplementary Table S4). In this model, the participants aged ≥45 years had statistically significant higher frequency of GP consultations, compared with the reference group of people aged 16–24 years, and the OR was increased from 1.48 to 2.54 for people aged ≥75 years.

Socioeconomic position was measured by three explanatory variables, QIMD, QEHI, and HEQ, but the final parsimonious model included QEHI only. Because these three socioeconomic variables are known to be strongly associated with each other, sensitivity analyses were conducted using either QIMD or HEQ instead of QEHI in the model. If it was used as a sole variable indicating socioeconomic position, the HEQ was statistically significantly associated with the times a GP was consulted (see Supplementary Table S5). If the QIMD was instead used, the most deprived were statistically significantly associated with increased GP consultations (OR 1.17, 95% CI = 1.02 to 1.36; *P* = 0.029), although the overall association between the QIMD and GP consultations was statistically non-significant (*P* = 0.302).

## Discussion

### Summary

It was found that the frequency of GP consultation was independently associated with sex, age, socioeconomic status, lasting illnesses, smoking, and body mass index. Although older age was associated with more frequent consultations for physical health problems, younger age was associated with more frequent consultations for any mental health problems. Lasting illnesses was the strongest predictor of consultation frequency, and much more so for consultations for mental than for physical health problems. The remarkably high OR between lasting illnesses and mental health consultation is likely to be both because conditions such as depression and anxiety are recognised by participants as lasting illnesses, and also because chronic physical conditions commonly have adverse effects on mental health.^12^

### Strengths and limitations

This study explored a broad range of factors associated with GP consultations, using data from HSE 2019, while previous studies usually used data from the CPRD.^1,3,13,14^ Some important factors considered in this study have not been investigated in previous studies. For example, HSE 2019 provided data on three socioeconomic variables, including Index of Multiple Deprivation, equivalised household income, and HEQ. In addition, previous studies have tended to focus on specific conditions, such as diabetes^15^ or mental health problems,^13^ and rarely explored the association between the GP consultation and the existence of lasting conditions in general. HSE 2019 data also allowed the study to distinguish consultations for physical and mental health problems.

This study has some limitations. All dependent and explanatory variables were categorical, which determined the statistical methods used. Ordered logistic regression analysis is the most appropriate analytic method for the ordinal outcome data in this study, and the results indicated relative differences (that is, ORs) in GP consultations between population groups. Further detailed numerical data on GP consultations are required to estimate the absolute differences in GP consultations between population groups.^10^ In addition, the GP consultations data did not record who initiated the consultation, consultations with general practice personnel other than GPs, had a lack of detailed data on reasons for consultations, and whether the consultation was face to face or remote by telephone. The data on the frequency of GP consultation and most other variables used in this study were self-reported, and thus likely to be approximate and subject to recall biases. HSE had a cross-sectional design, and it was not possible to elucidate causal pathways, such as the direction of effects between income, obesity, and lasting illnesses, and their effects on consultations. However, it is highly plausible that the independent variables included in the analyses were primarily causes rather than effects of consultation frequency. Finally, the HSE 2019 data were collected before the COVID-19 pandemic, and data from further studies are required to understand the current post-pandemic situation and to overcome the limitations of the study pointed out above.

### Comparison with existing literature

Findings from this study are mostly in line with those of previous studies, and consultation rates are higher among older patients, females, Asian ethnicity, and those who are socioeconomically disadvantaged.^3–5^ Compared with never smokers, former smoking was associated with more consultations for physical health problems, and current smoking tended to be associated with more consultations for any mental health problems. Although rurality is one of the determinants currently used for the NHS to allocate payments to general practice in England,^6^ a significant association between rurality and consultation frequency was not found.

Consultation rates in patients with mental health conditions were high in primary care.^13,16^ The contradictory directions of the association between age and the frequency of consultations for physical health problems and for any mental health problems has not to the authors' knowledge been reported previously. Consultations for any mental health problems were more likely among frequent users of GP consultations, females, younger adults, and those who are socioeconomically disadvantaged. The proportion of adults who consulted a GP for any mental health problems (16.6%) was smaller than those for physical health problems (83.5%). Because patients consulting a GP for any mental health problems were more likely to be frequent users. It could be estimated that nearly 25% of consultations were for patients with mental health or a combination of both mental and physical health problems. It is worth noting that the study precedes the COVID-19 pandemic, which clearly has impacted mental health for many.

The three socioeconomic variables are highly correlated with each other, so including them all in multivariable analyses would be likely to bias their association with the outcome. The final parsimonious multivariable models included QEHI only, indicating that household income is a better predictor of consultation frequency than the other two socioeconomic variables. However, each of the three socioeconomic variables are statistically significantly associated with the use of general practice, if only one of them was included in the models. This means that general practice needs to continue its aims to provide proactive and appropriate support for the material challenges that impact on people’s lives; for example, through social prescribing and community initiatives.^17^

Around 46% of adults reported health conditions lasting or expected to last ≥12 months. The existence of lasting illnesses was the strongest and most significant among explanatory variables for times consulted a doctor in the last 12 months. This reflects the real needs of patients for primary care. In addition, lasting illnesses may be a mediation variable of the causal path between other explanatory variables and consultation frequency. After excluding lasting illnesses from multivariable model, the association between older age and the consultation frequency became stronger. To a lesser extent, lasting illnesses may also mediate the association between the GP consultation and household income, smoking, and obesity.

### Implications for research and practice

The study has highlighted the importance of sex, age, ethnic group, socioeconomic inequality, long-term illness, smoking, overweight, and obesity as predictors of higher GP consultation rates. Age is not a readily modifiable risk factor, except by reducing mortality, and older age is associated with lower rates of mental health consultations. However, the prevalence and severity of long-term illness, socioeconomic inequality, smoking, overweight, and obesity are in principle modifiable through social and health policy. The results also show that long-term illness greatly increases the rate of consultations for mental health problems, suggesting that addressing the mental health of people with chronic physical conditions is an important need. Therefore, lowered prevalence of long-term illnesses in the population will reduce the needs for primary health care. The prevention of long-term conditions should start from early life, including school-aged children. Socioeconomic deprivation is clearly an important determinant of demand for general practice services, which should be taken into consideration in the NHS payments to general practices.^18^ Although in this study household income was more strongly associated with consultation frequency than area deprivation, area deprivation, based on census data, is more readily available than individuals’ household income.

In conclusion, the higher frequency of consultation of GPs is associated with female sex, older age, ethnic minority populations, socioeconomic disadvantage, existence of lasting illnesses, smoking, being overweight, and being obese. Older age is associated with increased consultations for physical health problems, but associated with reduced consultations for mental health or a combination of mental and physical health problems. The value of actively addressing personal background to problems at a systems level may help future demand management.

## Data

Health Survey for England 2019 data are available to view online: https://digital.nhs.uk/data-and-information/publications/statistical/health-survey-for-england/2019
